# Volunteer Conservation Action Data Reveals Large-Scale and Long-Term Negative Population Trends of a Widespread Amphibian, the Common Toad (*Bufo bufo*)

**DOI:** 10.1371/journal.pone.0161943

**Published:** 2016-10-05

**Authors:** Silviu O. Petrovan, Benedikt R. Schmidt

**Affiliations:** 1 Froglife, 1 Loxley, Werrington, Peterborough, PE45BW, United Kingdom; 2 Centre for Environmental and Marine Sciences, Scarborough Campus, University of Hull, Filey Road, Scarborough, YO11 3AZ, United Kingdom; 3 Department of Evolutionary Biology and Environmental Studies, University of Zurich, Winterthurerstrasse 190, 8057 Zurich, Switzerland; 4 KARCH, Passage Maximilien-de-Meuron 6, 2000 Neuchâtel, Switzerland; Vanderbilt University School of Medicine, UNITED STATES

## Abstract

Rare and threatened species are the most frequent focus of conservation science and action. With the ongoing shift from single-species conservation towards the preservation of ecosystem services, there is a greater need to understand abundance trends of common species because declines in common species can disproportionately impact ecosystems function. We used volunteer-collected data in two European countries, the United Kingdom (UK) and Switzerland, since the 1970s to assess national and regional trends for one of Europe’s most abundant amphibian species, the common toad (*Bufo bufo*). Millions of toads were moved by volunteers across roads during this period in an effort to protect them from road traffic. For Switzerland, we additionally estimated trends for the common frog (*Rana temporaria*), a similarly widespread and common amphibian species. We used state-space models to account for variability in detection and effort and included only populations with at least 5 years of data; 153 populations for the UK and 141 for Switzerland. Common toads declined continuously in each decade in both countries since the 1980s. Given the declines, this common species almost qualifies for International Union for the Conservation of Nature (IUCN) red-listing over this period despite volunteer conservation efforts. Reasons for the declines and wider impacts remain unknown. By contrast, common frog populations were stable or increasing in Switzerland, although there was evidence of declines after 2003. “Toads on Roads” schemes are vital citizen conservation action projects, and the data from such projects can be used for large scale trend estimations of widespread amphibians. We highlight the need for increased research into the status of common amphibian species in addition to conservation efforts focusing on rare and threatened species.

## Introduction

There is a current shift in conservation science away from the conservation of rare and endangered species towards the maintenance of ecosystem services [1]. Common species, while a small category in terms of species richness, can have a disproportionate impact in providing vital ecosystem structure and functions, primarily as a consequence of their overall biomass contribution [2, 3]. The major ecological significance of widespread and common species as well as the urgency in identifying, understanding, and halting their declines, has been repeatedly highlighted in recent years [2, 4–7]. Despite this, species conservation action and resources largely focus on rare species on the IUCN Red List. The rare species are frequently habitat specialists with small ranges. Common species often remain insufficiently monitored and neglected if declining at rates that do not make them regionally threatened and can in turn become generally rarer and extinct at the local level before prompting changes in policies [8]. Common or even abundant species that have declined dramatically in recent decades encompass a wide range of taxonomic groups, most notably moths, butterflies, fish and birds. They include species that are not directly exploited by humans but are impacted by the wide-ranging processes related to agricultural intensification and urbanisation [7, 9, 10]. However, despite the ongoing global amphibian decline [11, 12] and some recent progress in establishing new monitoring schemes, data on national- or continental-scale abundance trends of widespread and common amphibians remains particularly scarce.

Here, we use data from long-term and large-scale conservation action by volunteers to quantify population trends for one of Europe’s most common amphibians, the common toad, *Bufo bufo*. The common toad is a wide-ranging, adaptable and relatively abundant species listed as “least concern” by the IUCN [13] even though declines have been reported throughout its range [14–17]. The causes for these declines or their actual extent remain unclear but they are likely factors that operate at the landscape scale. In particular, habitat loss and fragmentation, including road impacts, and the effects of climate change have been blamed in the past [15, 16, 18, 19].

Similar to other amphibian species, toad populations can fluctuate widely between years, and sufficiently long time series are needed in order to quantify trends [20]. Also, common toads are mainly nocturnal, occupy a diverse range of habitats including farmland, are explosive breeders with a very short breeding season, and typically breed in vegetated areas of large bodies of water including lakes. As such, gathering data at the regional or national scale is logistically challenging. However, while robust abundance data at the regional or national scale for common amphibians are generally very scarce, such data are available for this species. Toads undertake large-scale spring breeding migrations, which often are intersected by roads and, consequently, the species typically represents the highest percentage of road-killed amphibians across much of Europe [21–23]. The plight of toads during annual mass mortality events on some roads [24, 25] has led to the establishment across Europe of “toad patrols” as early as the 1950s [22], where volunteers collect migrating amphibians and carry them across the road and towards the breeding site. Significantly, this has marked in many countries the beginning of conservation action for amphibians, has generated some of the earliest examples of citizen science data for this group, and toad patrols still represent one of the largest conservation actions undertaken by volunteers [26, 27]. Over 90,000 adult toads are currently carried across roads every year in some 160 sites by such volunteer groups in the UK (http://www.froglife.org/what-we-do/facts-figures/) and 700,000 amphibians (all species) are moved at more than 440 sites in Switzerland [22]. These sites, many of them established as early as the 1970s or 1980s, are widely distributed at a national scale in both rural and semi-urban settings. They encompass a broad range of toad population sizes, from less than 100 adult individuals to exceptional populations of 5,000–12,000 adults moved annually, therefore creating a valuable dataset for the analysis of population trends. Equally, other amphibian species are also moved at the same time by volunteers rescuing toads, especially common frogs *Rana temporaria*. Common frogs are a similarly widespread IUCN “least concern” species with “stable” trends, although some declines have been recorded in parts of their range [28]. Including this species in the analysis can therefore act as a control for trend estimation for common toads as well as indicate potential differences in responses to environmental change by the two species.

Knowing whether common species are stable or in decline is vital for directing conservation efforts and maintaining ecosystem services provided by amphibians [1, 29], yet these data are typically lacking. As stipulated by IUCN, one can use both distribution and abundance data to quantify population declines, and both have been used for amphibians [11, 17, 30]. We used volunteer-collected data as part of the “Toads on Roads” project coordinated and collated by Froglife in the UK and the amphibian migration database maintained by Koordinationsstelle für Amphibien- und Reptilienschutz in der Schweiz (KARCH) in Switzerland in order to identify and quantify long-term trends in population size (abundance) for these common species at national and regional scales. We hypothesised that such datasets can provide substantial information on historical trends. We estimated trends for different decades such that we could assess whether the rate of decline remained stable or changed through time.

## Methods

### Data collection

Data on numbers of toads and frogs moved by volunteers during spring migrations were collected at the end of the migration season which typically runs between March and April. Standard forms were completed by the patrol leader every night of the season (typically for 25–30 nights every year) collated into a season total, and sent off to be centralised. We used the seasonal total of toads and frogs moving towards the pond rather than the daily counts in the analysis. Volunteers collected and counted all the individual toads by repeatedly surveying a stretch of the road during each survey night. However, at some sites in the UK and most sites in Switzerland, temporary plastic fences were used in order to prevent toads from accessing the road, and the amphibians were then collected along the fences [22]. Given the timing, selected to coincide with the breeding season, the vast majority of the individuals recorded was represented by adults. Toads and other amphibians were collected in plastic buckets, taken to the other side of the road and released, often near the breeding area.

We used data from volunteer groups collected from 1975 to 2014 in the UK and from 1973 to 2012 in Switzerland (see Supporting Information), and we only included sites with at least 5 years of data so that enough years were included to remove the bias effects of natural population variations [20, 31, 32] and to establish a trend. Given the small number of populations between 1975 and 1984 for the UK, we used 1985–2014 data only. For the UK, we included a regional component by splitting the data into 5 regions following a similar previous analysis [15]. The populations were structured by county and separated into Northern, Western, Central, Eastern and South-Eastern. Regions are defined as follows: North (Scotland, Northumberland, Cumbria, Durham, Yorkshire, Lancashire, Cheshire and Derbyshire); West (Wales, Shropshire, Staffordshire, Herefordshire, Worcestershire, Gloucestershire, Wiltshire, Dorset, Somerset, Devon, Cornwall); Central (Nottinghamshire, Leicestershire, Northamptonshire, Rutland, Cambridgeshire, Warwickshire, Oxfordshire, Buckinghamshire, Bedfordshire); East (Lincolnshire, Norfolk, Suffolk, Essex, Hertfordshire); South-East (Berkshire, London, Hampshire, Surrey, Kent, Sussex, Isle of Wight). For Switzerland, we also included data on the abundance of the common frog (*Rana temporaria*) for comparison.

### Statistical analysis

After removing sites with less than 5 years of data, a total of 153 sites were included in the analysis for the UK and 141 for common toads and 135 for common frogs for Switzerland. The average length of time series for common toads in the UK was 14.1 years. In Switzerland, time series lengths for common toads and common frogs were 14.3 years and 14.0 years, respectively.

The data were collected by a large number of volunteers in many different places and years using similar but not identical methods. It is thus important to account for variation in sampling effort between sites and years [33]. Estimating detection probabilities and adjusting counts is a commonly used method to adjust for variation in sampling effort [34]. Therefore, we used the open N-mixture model for spatially replicated repeated count data [35] because this model is highly suitable for our data [36]. This model can be used to estimate population trends while accounting for variation in sampling effort and detectability because it assumes that the number of individuals is generally underestimated. The Dail-Madsen model was successfully employed for the analysis of similar amphibian population data [37–39].

Population trend is commonly estimated using a regression of abundance against calendar year [40–42]. We used population growth rate as our estimate of trend because we believe that it is a biologically more meaningful metric [43–45]. The model is [35, 36]

$$N_{i,t}∼\text{Poisson}(\lambda N_{i,t\text{-}1})$$ (eq 1)

where *N* is population size, *i* and *t* are indices for site and year, respectively, λ is population growth rate, and *N*_*i*,*t*_ is a Poisson random variable which allows for demographic stochasticity. Given this definition, the trend quantifies a proportional change in abundance (i.e., the population multiplication rate) rather than an absolute change. The biological trend model is related to the counts *y*_*i*,*t*_ using

$$y_{i,t}∼\text{Binomial}(p,N_{i,t})$$ (eq 2)

where *p* is detection probability [35, 36]. We fitted separate models for the Swiss und UK data and estimated parameters for both countries in independent analyses. For the purpose of our analysis, we estimated a mean λ per decade. Within the UK, we estimated a region- and decade-specific λ in a second analysis. Mean λ reduces the effects of random variation among years and sites. Detection probability *p* was allowed to vary among decades and regions (the latter only in the second analysis).

Model fitting followed the procedures outlined in [46]. We modified code available from the electronic supplement of Hostetler and Chandler [36] and fitted the models in the Bayesian software JAGS [47] using R and the R package jagsUI [48, 49]. To fit the models, we used a Poisson distribution to describe initial abundance *N*_*i*,1_. Because time series started in different years, we wrote the model in JAGS in such a way that *N*_*i*,*1*_ was replaced with *N*_*i*,*first t*_ where *first* was the first year of the time series *i*. We specified diffuse uniform priors for all parameters (*p*, λ and *N*_*i*,*first*_) to be as uninformative as possible. For each model, we ran three Markov chains with 10000 iterations each, discarded the first 2000 iterations as burn-in and thinned the remainder by one in ten. Convergence was assessed using the Brooks–Gelman–Rubin $\hat{R}$ statistic [46].

## Results

**Fig 1:**
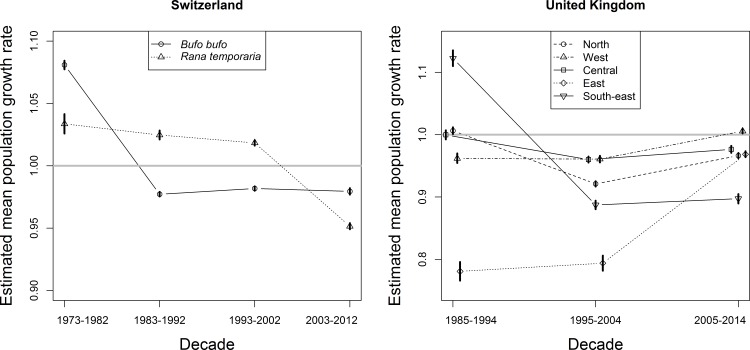
Estimated mean population growth rates. Estimates (and 95% credible intervals; some intervals are smaller than the symbols) are shown for four decades for the common toad (*Bufo bufo*) and the common frog (*Rana temporaria*) in Switzerland (left panel) and for three decades for the common toad (*Bufo bufo*) in five regions of the United Kingdom (right panel).

Detectability varied among countries, regions, decades and species from 0.198 to 0.773 (Table 1). Overall, population growth rates were negative for most decades in both Switzerland and the UK (Fig 1), implying that toad abundance declined in both countries since the mid-1980s. In the UK, the mean population growth rates for the decades 1985 to 1994, 1995 to 2004 and 2005 to 2014 were 0.989 (95% CRI: 0.985, 0.992), 0.929 (95% CRI: 0.927, 0.932) and 0.973 (95% CRI: 0.971, 0.975), respectively (Fig 1). For the UK, where regional analysis was included, toads declined in almost all regions and all decades, although declines seemed to become reversed in the 2005–2014 decade in region West, encompassing Wales and South-West England. The most pronounced declines in the UK were in South-East England, while in the Eastern region, comprising mostly East Anglia, trend estimations indicated that initial severe declines in previous decades were followed by a recovery since 2005, although not enough to reverse the overall decline (Fig 1).

**Table 1:** Estimated detection probabilities (and 95% credible intervals).

Country	Species	Region	Decade			
				1985–1994	1995–2004	2005–2014
UK	*Bufo bufo*	North		0.233	0.402	0.506
				(0.226, 0.240)	(0.393, 0.410)	(0.494, 0.517)
		West		0.304	0.379	0.470
				(0.293, 0.316)	(0.371, 0.389)	(0.462, 0.478)
		Central		0.234	0.373	0.525
				(0.227, 0.240)	(0.365, 0.381)	(0.511, 0.538)
		East		0.198	0.350	0.455
				(0.192, 0.205)	(0.328, 0.372)	(0.441, 0.470)
		South-east		0.557	0.541	0.773
				(0.536, 0.579)	(0.523, 0.557)	(0.756, 0.790)
			1973–1982	1983–1992	1993–2002	2003–2012
Switzerland	*Bufo bufo*		0.390	0.358	0.420	0.443
			(0.384, 0.395)	(0.355, 0.362)	(0.416, 0.424)	(0.438, 0.449)
	*Rana temporaria*		0.362	0.406	0.323	0.434
			(0.352, 0.373)	(0.400, 0.411)	(0.320, 0.327)	(0.428, 0.439)

In Switzerland, the mean population growth rates for common toad (*Bufo bufo*) populations for the four decades 1973 to 1982, 1983 to 1992, 1993 to 2002 and 2003 to 2012 were 1.081 (95% CRI: 1.077, 1.084), 0.977 (95% CRI: 0.975, 0.979), 0.982 (95% CRI: 0.980, 0.984), and 0.980 (95% CRI: 0.977, 0.982), respectively (Fig 1). The corresponding values for the common frog populations (*Rana temporaria*) were 1.034 (95% CRI: 1.026, 1.041), 1.025 (95% CRI: 1.021, 1.028), 1.018 (95% CRI: 1.016, 1.021) and 0.925 (95% CRI: 0.949, 0.954).

## Discussion

### Volunteer conservation action as a long-term monitoring tool

Citizen science projects have been gaining attention due to their potential for collecting large amounts of data over wide spatial and temporal scales as well as a means of engaging the public in conservation efforts. Importantly however, the primary motivation for “toad patrols” was the conservation action of protecting amphibians from road mortality rather than surveys or data collection, making this dataset relatively unique as volunteers collect data measuring not just the number of animals present but also their own success in moving them to safety. Expanding such schemes that provide both highly valuable monitoring data and a measurable conservation action that attracts and engages people could be a successful model for other citizen science wildlife projects [50]. Cooke [19] demonstrated that the decline in numbers of toads recorded killed on roads at three unpatrolled sites in the UK between 1974 and 2010 correlated strongly with numbers of adults reaching the ponds, making the numbers of individuals at road crossing points a suitable measure of population abundance. In our analysis, we used statistical models to account for imperfect detection and variation in sampling effort. Detection probabilities were sometimes low but this may be an effect of the structure of the model. If repeated counts within years were available, we might have been able to model detectability in a better way [36]. Allowing for environmental stochasticity in the models [36] might lead to higher estimates of detection probabilities because the model may be better able to disentangle variation in detection and natural population fluctuations.

### Road-based surveys for national-scale abundance trends estimations for common amphibians

The selection of our sites was not random but rather volunteers chose them based on observing amphibian mortality on specific road sections, and as such, it is unclear how representative they are for toad populations at a national scale in each country. However, declines of common toads (based on presence/absence data) in Switzerland were also independently found in a survey where sites were selected randomly [17]. There are two reasons why we believe that the studied populations are representative nationally and important. First, roads are known to have strong negative effects on amphibian populations [51]. Both the UK and Switzerland have some of the densest and most intensely used road networks in the world when compared to the total surface area of the country [52]. For example, Switzerland has a road density of 2.7 km/km^2^ [22] and considerably higher in the lowlands. Similar to other Western or Central European countries, given the high habitat fragmentation degree in both the UK and Switzerland, there are probably few areas other than mountains where toad populations migrating 1 km or more towards the breeding area would not have to cross at least one road. Thus, it seems likely that the majority of UK and Swiss toad populations are affected to some extent by roads. Second, even though the sample is not random, this dataset represents hundreds of populations that benefit from conservation action, and one might thus expect that they fare better than other populations with no such conservation action.

### Significant and continuous declines of common toads since 1980s

An annual trend of 0.96 implies that a population will decline by more than 30% in less than ten years. Given this magnitude of decline, a population would qualify as “vulnerable” under IUCN Red List criterion A2 (“Population reduction observed, estimated, inferred, or suspected in the past where the causes of reduction may not have ceased OR may not be understood OR may not be reversible.”). The observed declines in our dataset were close to this figure or even surpassed it at both regional level and national level (common toads in the UK between 1995 and 2004). The fact that toad abundance significantly declined across both countries since the 1980s even at sites where there was an obvious and long term conservation action in place, (i.e. moving individual toads across the roads by volunteers) is troubling. Our results appear to confirm that such actions are not sufficient to prevent long-term declines, as previously observed for 33 toad populations in Italy [16]. Reasons for the decline of the common toad might include the fact that volunteers in “Toads on Roads” projects only target adult migration towards the wetland but not the unpredictable adult return migration or the movement of juveniles dispersing from the breeding area in late summer. Thus, the postmetamorphic juvenile stage, whose survival is crucial for amphibian population dynamics [53–55], is not protected from road mortality [22]. Road traffic and car numbers have increased substantially in both countries since 1980, almost doubling in the UK within that period to 35 million vehicles registered in 2013 [56], potentially making it increasingly difficult for juvenile toads moving over the road to escape car traffic. Thus, large and exceptional amphibian populations should be protected from road impacts and habitat loss by adequate mitigation such as the construction of underpasses rather than solely through such volunteer actions. However, questions remain about the effectiveness of toad tunnels for juveniles [22].

Additionally, road mortality represents only one potential cause of decline, and other factors such as climate change and increasingly mild winters [18], agricultural intensification and widespread habitat loss or degradation, as well as emergent diseases [57], remain unresolved and ongoing and may affect toads both in their aquatic and terrestrial habitats. The fact that toad declines appeared most pronounced in South-East England, the most densely populated region in the UK, is important and requires further investigation. Similarly, the steep decline in Switzerland since the 1980s might be linked with both road traffic increases, changing climate, and widespread habitat degradation as already observed in declines of other widespread species. A potential solution to halt declines would be to urgently incorporate widespread and common species such as toads into wider conservation and planning policy initiatives and target large-scale improvements of the general landscape using networks of agri-environment scheme options and landscape-scale connectivity projects.

It is likely that in the absence of volunteer efforts toad declines would have been more marked and more populations would have become extinct given the unsustainable levels of adult mortality, as road mortality can drive toad populations to extinction [58]. This was the case at 3 sites monitored in Cambridgeshire, UK where no animals were moved by volunteers and instead effort concentrated on annual recording of toad mortality and traffic levels on the road from 1974 until all three populations collapsed by 2010 [19]. Local extinction sites are almost certainly under-represented in our datasets as volunteers may become disheartened following prolonged declines and give up patrolling the site once the numbers are low.

Data collected partly from the same period seems to indicate that for the other widespread and abundant amphibian species, the common frog, populations appear to have stabilised in both Switzerland and the UK ([15, 17], this study), although our analysis indicates recent declines in Switzerland. The reasons for this disparity in trends are unknown. Carrier and Beebee [15] suggested that common frogs are better adapted to breed in shallow and small bodies of water, including garden ponds, which seem to have provided important refuge for the species in the face of lost habitats in farmland areas. It is, however, also possible that common frogs are less impacted at the population level by road mortality either for juveniles only or for both juveniles and adults. Understanding why one common generalist species declines while the other appears to be stable would be an important focus for future research.

### Common species continue to decline in Europe but largely remain neglected

Despite some recent increase in interest in the value of common species and the importance of their declines [4], conservation resources, including monitoring as well as government policy at a national level, remain almost entirely focused on rare species which are facing regional or global extinctions in the near future. Both common birds and common butterfly species, including former pest species, are declining in Europe while effective conservation programs for habitat specialists mean that at least some of the rarer species are increasing in abundance [7,9]. However, commonness itself is rare, and even moderate declines in a small number of common species can inherently mean the loss of vast numbers of individuals and biomass over large areas with consequences across the trophic chain, habitat or ecosystem function [2, 10]. Common toads for example, are important invertebrate predators and also a regular food source for a wide range of species in Europe, including mammals, reptiles and birds (for a review on ecosystem functions of amphibians see Hocking and Babbitt [29].

## Conclusions

The long-term and ongoing decline of one of Europe’s most common amphibians is a significant cause for concern and has unknown wider impacts. Yet, this decline has been uncovered almost incidentally given the lack of specific long-term monitoring data for common amphibians. We demonstrate that “toad patrol” schemes, the earliest and largest examples of conservation action for amphibians, can produce robust datasets for trend estimation but require long-term data and adequate analysis, and we urge other countries to use such existing datasets in similar ways. The timing and similarity of the toad declines in both countries suggests a potential commonality of causes, but the exact reasons for the declines remain currently unknown. The recent declines of common species (e.g., birds and butterflies [7,9]) in Western Europe could be explained by the fact that common species occupy areas of land mostly outside of protected areas, including for the common toad, farmland and semi-urban areas. Their declines could be linked to the general deterioration and fragmentation of the quality of the environment on a landscape scale and which cannot be offset by smaller improvements elsewhere, such as in well managed reserves [2, 59]. Thus, the spatial scale at which declines of common species are observed suggests a large-scale deterioration of environmental quality.

While significant conservation improvements have recently been achieved for some endangered species [59], common species, including amphibians, are still rapidly declining in Europe, largely unnoticed due to lack of resources for monitoring and despite the fact that such species have a disproportionate impact in providing ecosystem function and structure. Although conservation goals have moved towards a more wide-encompassing approach that incorporates ecosystem goods and services [1], this requires a shift in conservation practice that makes it clear that it is not sufficient to protect habitats of rare specialists. Conservation efforts need to focus more on generalist widespread and common species and the countryside as a whole if system function and resilience are to be maintained.

## Supporting Information

S1 File Common toad time series data from Switzerland. The file contains the raw numbers of toads that were counted.
(TXT)

S2 File Common frog time series data from Switzerland. The file contains the raw numbers of toads that were counted.
(TXT)

S3 File Common toad time series data from the UK. The file contains the raw numbers of toads that were counted.
(TXT)

## References

[1] Mace GM. Whose conservation? Science 2014; 345: 1558–1560. 10.1126/science.1254704 25258063

[2] Gaston KJ, Fuller RA. Commonness, population depletion and conservation biology. Trends Ecol Evol. 2008; 23: 14–19. 18037531 10.1016/j.tree.2007.11.001

[3] Kleijn D, Winfree R, Bartomeus I, Carvalheiro LG, Henry M, Isaacs R, et al Delivery of crop pollination services is an insufficient argument for wild pollinator conservation. Nat Commun. 2015; 6 10.1038/ncomms8414 PMC4490361 26079893

[4] Gaston KJ. Valuing common species. Science 2010; 327: 154–155. 10.1126/science.1182818 20056880

[5] Lindenmayer DB, Wood JT, McBurney L, MacGregor C, Youngentob K, Banks SC. How to make a common species rare: a case against conservation complacency. Biol Conserv. 2011; 144: 1663–1672.

[6] Redford KH, Berger J, Zack S. Abundance as a conservation value. Oryx 2013; 47: 157–158.

[7] Inger R, Gregory R, Duffy JP, Stott I, Voříšek P, Gaston KJ. Common European birds are declining rapidly while less abundant species' numbers are rising. Ecol Lett. 2015; 18: 28–36. 10.1111/ele.12387 25363472

[8] Kamp J, Oppel S, Ananin AA, Durnev YA, Gashev SN, Hölzel N, et al Global population collapse in a superabundant migratory bird and illegal trapping in China. Conserv Biol. 2015; 29: 1684–1694. 10.1111/cobi.12537 26059233

[9] Van Dyck H, Van Strien AJ, Maes D, Van Swaay CA. Declines in common, widespread butterflies in a landscape under intense human use. Conserv Biol. 2009; 23: 957–65. 19637406 10.1111/j.1523-1739.2009.01175.x

[10] Gaston KJ. Common ecology. BioScience. 2011; 61: 354–362. 10.1525/bio.2011.61.5.4

[11] Houlahan JE, Findlay CS, Schmidt BR, Meyer AH, Kuzmin SL. Quantitative evidence for global amphibian population declines. Nature 2000; 404: 752–755. 10783886 10.1038/35008052

[12] Stuart SN, Chanson JS, Cox NA, Young BE, Rodrigues AS, Fischman DL, et al Status and trends of amphibian declines and extinctions worldwide. Science 2004; 306: 1783–1786. 15486254 10.1126/science.1103538

[13] Agasyan A, Avisi A, Tuniyev B, Isailovic JC, Lymberakis P, Andrén C, et al. 2009. Bufo bufo. The IUCN Red List of Threatened Species 2009: e.T54596A11159939. Available from 10.2305/IUCN.UK.2009.RLTS.T54596A11159939.en (accessed February 2016).

[14] Cooke AS. Indications of recent changes in status in the British Isles of the frog (*Rana temporaria*) and the toad (*Bufo bufo*). J Zool. 1972; 167: 161–178.

[15] Carrier JA, Beebee TJ. Recent, substantial, and unexplained declines of the common toad *Bufo bufo* in lowland England. Biol Conserv. 2003; 111: 395–399.

[16] Bonardi A, Manenti R, Corbetta A, Ferri V, Fiacchini D, Giovine G, et al Usefulness of volunteer data to measure the large scale decline of “common” toad populations. Biol Conserv. 2011; 144: 2328–2334.

[17] Cruickshank SS, Ozgul A, Zumbach S, Schmidt BR. Quantifying population declines based on presence‐only records for Red List assessments. Conserv Biol. 2016; 30, in press. 10.1111/cobi.12688 26864587

[18] Reading CJ. Linking global warming to amphibian declines through its effects on female body condition and survivorship. Oecologia 2007; 151: 125–131. 17024381 10.1007/s00442-006-0558-1

[19] Cooke AS. The role of road traffic in the near extinction of Common Toads (*Bufo bufo*) in Ramsey and Bury. NatCambridgesh. 2011; 53: 45–50.

[20] Green DM. The ecology of extinction: population fluctuation and decline in amphibians. Biol Conserv. 2003; 111: 331–343.

[21] Hels T, Buchwald E. The effect of road kills on amphibian populations. Biol Conserv. 2001; 99: 331–340.

[22] Schmidt BR, Zumbach S. Amphibian road mortality and how to prevent it: a review. Urban herpetology. Herpetol Conserv. 2008; 3: 157–167.

[23] Hartel T, Moga CI, öllerer K, Puky M. Spatial and temporal distribution of amphibian road mortality with a *Rana dalmatina* and *Bufo bufo* predominance along the middle section of the Târnava Mare basin, Romania. North-West J Zool. 2009; 5: 130–41.

[24] Meisterhans K, Heusser H. Amphibien und ihre Lebensräume: Gefährdung, Forschung, Schutz. Naturforschende Gesellschaft, Naturschutzkommission; 1970.

[25] Van Gelder JJ. A quantitative approach to the mortality resulting from traffic in a population of *Bufo bufo* L. Oecologia 1973; 13: 93–95. 28307986 10.1007/BF00379622

[26] Langton TE. Amphibians and roads; proceedings of the Toad Tunnel Conference, Rendsburg, Federal Republic of Germany, 7–8 January 1989.

[27] Langton TES. A history of small animal road ecology In: Andrews KM, Nanjappa P, Riley SP, editors. Roads and ecological infrastructure: concepts and applications for small animals. Johns Hopkins University Press, Baltimore, USA; 2015 pp. 7–19.

[28] Kuzmin S, Ishchenko V, Tuniyev B, Beebee T, Andreone F, Nyström P, et al. 2009. Rana temporaria. The IUCN Red List of Threatened Species 2009: e.T58734A11834246. 10.2305/IUCN.UK.2009.RLTS.T58734A11834246.en. Downloaded on 10 June 2016

[29] Hocking DJ, Babbitt KJ. Amphibian contributions to ecosystem services. Herpetol Conserv Biol. 2014; 9: 1–7.

[30] Grant EHC, Miller DAW, Schmidt BR, Adams MJ, Amburgey SM, Chambert T, et al Quantitative evidence for the effects of multiple drivers on continental-scale amphibian declines. Sci Rep. 2016; 6: 25625 10.1038/srep25625 27212145 PMC4876446

[31] Marsh DM. Fluctuations in amphibian populations: a meta-analysis. Biol Conserv. 2001; 101: 327–335.

[32] Meyer AH, Schmidt BR, Grossenbacher K. Analysis of three amphibian populations with quarter–century long time–series. ProcR Soc London B: Biol Sci. 1998; 265: 523–528. 10.1098/rspb.1998.0326 PMC1688910 9606133

[33] Schmidt BR. Count data, detection probabilities, and the demography, dynamics, distribution, and decline of amphibians. C R Biol. 2003; 326: S119–S124. 14558460 10.1016/s1631-0691(03)00048-9

[34] Kéry M, Schmidt BR. Imperfect detection and its consequences for monitoring for conservation. Comm Ecol. 2008; 9: 207–216.

[35] Dail D, Madsen L. Models for estimating abundance from repeated counts of an open metapopulation. Biometrics 2011; 67: 577–587. 10.1111/j.1541-0420.2010.01465.x 20662829

[36] Hostetler JA, Chandler RB. Improved state‐space models for inference about spatial and temporal variation in abundance from count data. Ecology 2015; 96: 1713–1723.

[37] Hocking DJ, Babbitt KJ, Yamasaki M. Comparison of silvicultural and natural disturbance effects on terrestrial salamanders in northern hardwood forests. Biol Conserv. 2013; 167: 194–202.

[38] Mazerolle MJ, Perez A, Brisson J. Common reed (*Phragmites australis*) invasion and amphibian distribution in freshwater wetlands. Wetlands Ecol Manag. 2014; 22: 325–340.

[39] Zylstra ER, Steidl RJ, Swann DE, Ratzlaff K. Hydrologic Variability Governs Population Dynamics of a Vulnerable Amphibian in an Arid Environment. PLoS ONE. 2015; 10: e0125670 10.1371/journal.pone.0125670 26030825 PMC4452645

[40] Wade PR. Bayesian methods in conservation biology. Conserv Biol. 2000; 14: 1308–1316.

[41] Dixon PM, Pechmann JHK. A statistical test to show negligible trend. Ecology 2005; 86: 1751–1756.

[42] Schmidt BR, Meyer AH. On the analysis of monitoring data: Testing for no trend in population size. J Nat Conserv. 2008; 16: 157–163.

[43] Tobler U, Borgula A, Schmidt BR. Populations of a susceptible amphibian species can grow despite the presence of a pathogenic chytrid fungus. PLoS ONE. 2012; 7: e34667 10.1371/journal.pone.0034667 22496836 PMC3320643

[44] Doddington BJ, Bosch J, Oliver JA, Grassly NC, Garcia G, Schmidt BR, et al Context-dependent amphibian host population response to an invading pathogen. Ecology 2013; 94: 1795–1804. 24015523 10.1890/12-1270.1

[45] Buckley J, Beebee TJC, Schmidt BR. Monitoring amphibian declines: population trends of an endangered species over 20 years in Britain. Anim Conserv. 2014; 17: 27–34.

[46] Kéry M, Schaub M. Bayesian population analysis using WinBUGS: a hierarchical perspective Academic Press; 2012.

[47] Plummer M. JAGS: A program for analysis of Bayesian graphical models using Gibbs sampling. InProceedings of the 3rd international workshop on distributed statistical computing 2003 Mar 20 (Vol. 124, p. 125). Wien, Austria: Technische Universität Wien.

[48] R Core Team. R: A language and environment for statistical computing R Foundation for Statistical Computing, Vienna, Austria 2014 http://www.R-project.org/

[49] Kellner, K: jagsUI: A Wrapper Around 'rjags' to Streamline 'JAGS' Analyses. R package version 1.3.7. Available from http://CRAN.R-project.org/package=jagsUI (accessed August 2015).

[50] Lawson B, Petrovan SO, Cunningham AA. Citizen Science and Wildlife Disease Surveillance. EcoHealth 2015; 12: 693–702. 10.1007/s10393-015-1054-z 26318592

[51] Cosentino BJ, Marsh DM, Jones KS, Apodaca JJ, Bates C, Beach J, Beard KH, et al Citizen science reveals widespread negative effects of roads on amphibian distributions. Biol Conserv. 2014; 180: 31–38.

[52] OECD. Road traffic, vehicles and networks Environment at a glance 2013: OECD Indicators, OECD Publishing 10.1787/9789264185715-en

[53] Lampo M, De Leo GA. The invasion ecology of the toad *Bufo marinus*: from South America to Australia. Ecol Appl. 1998; 8: 388–96.

[54] Hels T, Nachman G. Simulating viability of a spadefoot toad *Pelobates fuscus* metapopulation in a landscape fragmented by a road. Ecography 2002; 25: 730–44.

[55] Di Minin E, Griffiths RA. Viability analysis of a threatened amphibian population: modelling the past, present and future. Ecography 2011; 34: 162–169.

[56] Department for Transport. Vehicle Licensing Statistics 2013. Statistical release 10 April 2014. National Statistics. https://www.gov.uk/government/statistics/vehicle-licensing-statistics-2013

[57] Price SJ, Garner TW, Nichols RA, Balloux F, Ayres C, de Alba AM, et al Collapse of amphibian communities due to an introduced Ranavirus. Curr Biol. 2014; 24: 2586–91. 10.1016/j.cub.2014.09.028 25438946

[58] Heusser H. Wie Amphibien schützen? Flugblatt Naturforschende Gesellschaft Schaffhausen. 1968; 3: 1–14.

[59] Hoffmann M, Hilton-Taylor C, Angulo A, Böhm M, Brooks TM, Butchart SHM, et al The impact of conservation on the status of the world’s vertebrates. Science 2010; 330: 1503–1509. 10.1126/science.1194442 20978281

